# Cobalt Chromium Molybdenum Surface Modifications Alter the Osteogenic Differentiation Potential of Human Mesenchymal Stem Cells

**DOI:** 10.3390/ma13194292

**Published:** 2020-09-25

**Authors:** Birgit Lohberger, Nicole Eck, Dietmar Glaenzer, Helga Lichtenegger, Leon Ploszczanski, Andreas Leithner

**Affiliations:** 1 Department of Orthopedics and Trauma, Medical University Graz, 8036 Graz, Austria; nicole.eck@medunigraz.at (N.E.); dietmar.glaenzer@medunigraz.at (D.G.); andreas.leithner@medunigraz.at (A.L.); 2 Department of Material Sciences and Process Engineering, Institute of Physics and Materials Science, University of Natural Resources and Life Sciences, 1160 Vienna, Austria; helga.lichtenegger@boku.ac.at (H.L.); leon.ploszczanski@boku.ac.at (L.P.)

**Keywords:** CoCrMo alloy, mesenchymal stem cells, osteogenic differentiation, bone biology, cpTi

## Abstract

Surface roughness on orthopedic implant materials has been shown to be highly influential on the behavior of osteogenic cells. Mesenchymal stem and progenitor cells (MSPCs) migrate to the interface, adhere, proliferate, and differentiate into osteoblasts, which subsequently form bone matrix. Modifications of the implant surfaces should accelerate this process and improve biocompatibility. In this study, five surface topographies on cobalt chromium molybdenum (CoCrMo) were engineered to examine the influence on MSPCs. Scanning electron microscopy revealed significant differences in the morphology of untreated CoCrMo discs in comparison with CoCrMo with a titanium nitride (TiN) coating, polished and porous coated CoCrMo surfaces, and CoCrMo with a pure titanium (cpTi) coating. Elemental analysis was performed using energy-dispersive X-ray spectroscopy (EDX). Human primary MSPCs were expanded from tissue samples of spongiosa bone and characterized according to the criteria of the International Society for Cellular Therapy. The characteristic phenotype of MSPC was confirmed by flow cytometry and multilineage differentiation. Alcaline phosphatase and osteopontin expression increased significantly in all groups about 5-fold and 10-fold, respectively, in comparison to the undifferentiated controls. The porous coated surface showed a reduced expression of osteogenic markers. Due to the osteogenic differentiation, the expression of integrin α5β1, which is particularly important for cell-material contact, increased 4–7-fold. In the dynamic process of bone biology, MSPCs cultured and differentiated on cpTi, showed significant upregulation of IL6 and leptin.

## 1. Introduction

Cobalt chromium molybdenum (CoCrMo) alloys are a class of bioactive materials widely used in orthopedic implants such as artificial hip and knee joints due to their corrosion resistance and excellent mechanical properties [1]. The osseointegration of prostheses in humans requires a period of several months [2]. Various factors such as implant design and implant material, surgical technique, biomechanical forces, and patient variables are responsible for the osseous integration of the implant. Surface modification techniques such as surface plasma-spraying, polishing, sandblasting, acid etching, and bioactive coatings have been identified as a potential approach to improve osseointegration [3,4,5]. In this study, modifications were made to a commercially available CoCrMo surface using various coating methods like a mechanical polishing process, sintering, plasma spraying, and physical vapor deposition (PVD). Titanium nitride (TiN) coating was carried out with PVD, which is an effective way to enhance the biocompatibility of metal implants [6]. Improved corrosion resistance and osseointegration and reduced ion release was achieved by coatings using plasma spray technology with pure titanium (cpTi) [7,8].

The biological targets of new implant surfaces are often mesenchymal stem and progenitor cells (MSPCs) and cells of the osteoblastic line [9]. These cells migrate to the interface, adhere, proliferate and differentiate into osteoblasts, which form bone matrix [10,11]. To accelerate and improve the process of osseointegration, osteoblast differentiation and matrix production should be stimulated and activated by the coating of the implant and its surface [12].

The osteogenic differentiation process takes place in several steps, whereby the osteoblasts express collagen type I, osteocalcin (OC), osteopontin (SPP1), and alkaline phosphatase (ALP) during the formation of the extracellular matrix. OC is the best known late stage marker of osteogenesis and reaches its expression maximum at the beginning of mineralization [13]. For the osteogenic differentiation of primary pluripotent MSPCs, standard cell culture protocols require the addition of special agents such as ascorbic acid and dexamethasone [14]. Dexamethasone primarily promotes cell proliferation whereas ascorbic acid induces the expression of ALP and OC [15]. Primary cells do not differentiate synchronously in vitro, since cell differentiation depends mainly on the place of cell collection, the methods of cell extraction and purification, the age and sex of the donor [16]. The current literature does not indicate which types and sizes of surface microtopography are most appropriate to promote the production of bone matrix. In osseointegration, metal ions play an important role in angiogenesis, osteogenesis, and mineralization of bone tissue. Several studies have been published on the effects of metal ions on different cellular systems [17]. The interactions between MSPCs and material surfaces occur mainly via integrins, which act as important cell receptors for the extracellular environment and play a central role in the adhesion and distribution of cells on material surfaces [18,19]. According to the current state of knowledge, integrin α5β1 is one of the few integrins that demonstrably participate in MSPC osteogenesis [19,20].

The aim of this study is to improve our knowledge of osteoinductive performance on different CoCrMo surfaces and the associated osteogenic and inflammatory mechanisms. We investigated the effect of titanium nitride (TiN) coating, a porous coated surface, a polished surface, and a coating with pure titanium (cpTi) on a CoCrMo alloy regarding the osteogenesis of primary human MSPCs, the expression of 13 osteogenic and inflammatory markers, and the changes of the integrin α5β1 state.

## 2. Materials and Methods

### 2.1. CoCrMo Alloy Surface Modifications

Implantcast (Buxtehude, Germany) produced the CoCrMo discs in a precision casting process according to the ISO 5832-4 specification. These contain the wrought CoCrMo alloy 59–65% Co, 26.8–30% Cr, 4.5–7% Mo, and less than 1% Ni, Fe, C, Si, and Mn. With the porous coated CoCrMo surface, three layers of 250–355 µm large spheres were created by sintering onto the original alloy, resulting in a layer thickness of 700–1060 µm and a porosity of 30–40%. The tensile strength was >34.5 MPa (pull off test), and the shear strength was >20 MPa (shear test). The ceramic surface coating with titanium nitride (TiN) is an additive process in which the coating is anchored to the implant surface and is therefore particularly wear-reducing, anti-allergic, and biocompatible. Using physical vapor deposition, titanium atoms are released from a solid target by electrical energy, ionized, and accelerated onto the implant surface, where they combine with nitrogen molecules to form TiN. The result is a 5.5 ± 1.5 µm thick golden yellow ceramic TiN layer with a mean surface roughness (Ra) <0.05 µm and an adhesive tensile strength >22 MPa. The coating with pure titanium (cpTi) by vacuum plasma spraying resulted in a rough and porous surface layer of 300 ± 50 µm thickness and a porosity of 30 ± 10%. The average surface roughness (Ra) was 50 ± 15 µm, the tensile strength >22 MPa, and the shear strength >20 MPa. All materials were manufactured with 14 mm diameter and 1 mm thick discs to fit 24-well cell culture plates. Sterilization with gamma radiation was performed according to the standardized protocols.

### 2.2. Scanning Electron Microscopy (SEM)

SEM and EDX measurements have been performed in a previous study [21], as briefly described follows. The SEM images were taken with the FEI Quanta 250 FEG (Thermo Fisher Scientific, Hillsboro, OR, USA) under high vacuum conditions and 20 kV high voltage. The micrographs were taken with an Everhart–Thornley detector in secondary electron (SE) mode. To ensure sufficient electrical conductivity, the surfaces were sputter-coated with a 10 nm thin gold layer. The energy-dispersive X-ray spectroscopy (EDX) measurements were performed for 60 s, 20 kV high tension, and a spot size of 4.5 with a 30 mm^2^ Octane Elect Plus Silicon Drift Detector by EDAX Ametek (Berwyn, PA, USA) and APEX standard software V1.3.1 from 6 July, 2019.

### 2.3. Intraoral Tissue Harvest and Cell Culture

During routine maxillofacial surgery, tissue samples of cancellous bone were taken and primary human mesenchymal stem and progenitor cells (MSPCs) were obtained using explanted cultures. The study protocol was approved by the local ethics committee, and patient informed consent was obtained (29-156ex16/17). A total of seven female patients, aged between 25 and 35, were included in the study. Bone samples were between 4 and 6 mm long with cortical or cortical-cancellous structure and were rinsed with phosphate-buffered saline. The biopsies were then transferred into culture flasks with growth medium and incubated for cell expansion in a humidified atmosphere with 5% CO_2_ at 37 °C. The following medium was used: *α*-modified minimum essential medium (*α*-MEM; Sigma-Aldrich, Vienna, Austria) supplemented with 10% human platelet lysate (HPL), 2 U/mL stabilizer-free heparin (Biochrom AG, Berlin, Germany), 2% penicillin streptomycin, 0.5% L-glutamine, 0.2% amphotericin B (GIBCO Invitrogen, Darmstadt, Germany), and 2.5% ((4-(2-hydroxyethyl)-1-piperazineethanesulfonic acid; HEPES) buffer (Sigma-Aldrich, Vienna, Austria).

### 2.4. Flow Cytometry

The monoclonal antibodies CD73 PE, CD90 APC, CD105 PE, CD45 APC-Cy7, CD34 APC, CD14 FITC, CD19 APC, and HLA-DR APC (BD Bioscience, San Jose, CA, USA) were used to characterize the MSPCs. The background staining for antibodies was performed on the control cell lines and with fluorochrome conjugated isotype controls. The flow cytometry analysis was performed on a FACS LSRII system and the FACSDiva software (BD Bioscience, San Jose, CA, USA). For the evaluation, the FCS Express software (DeNovo Software, Los Angeles, CA, USA) was used. Using forward scatter (FSC) and side scatter (SSC), cell aggregates and dead cells were excluded. Data from all donors were analyzed under identical parameters and by collecting 10,000 events.

### 2.5. Multilineage Differentiation Analysis

The MSPC expansion medium consists of Dulbecco’s modified Eagle medium (DMEM-F12) expansion medium containing 10% foetal bovine serum (FBS), 1% penicillin streptomycin, 1% L-glutamine, and 0.1% amphotericin B; the osteogenic differentiation medium was supplemented with 100 nM dexamethasone, 0.1 mM ascorbic acid 2-phosphate, and 10 mM β glycerophosphate (all Sigma Aldrich). On days 7 and 14, the alkaline phosphatase (ALP) activity was determined by histochemical staining (ALP Kit No. 85; Sigma Aldrich, Vienna, Austria), according to the manufacturer’s instructions. ALP enzyme activity was determined by measuring the absorbance of the p-nitrophenol phosphate product formed at 405 nm on a microplate reader (BioRad Laboratories Inc., Hercules, CA, USA). Adipogenic differentiation was triggered by the addition of 100 nM dexamethasone, 50 µM indomethacin (Sigma Aldrich, Vienna, Austria), and 0.135 IU/mL insulin (Novo Nordisk, Bagsværd, Denmark). The adipocyte-specific fat vacuoles were detected by oil red O staining on day 21. The chondrogenic differentiation medium contained DMEM-F12 supplemented with 10% FBS, 1 ng/mL TGF-β3, and 100 µM L-ascorbic acid. After 21 days of chondrogenic induction the production of glycosaminoglycans and mucopolysaccharides was verified by Alcian blue staining. The cells were fixed with 10% formaldehyde and stained with 1% Alcian blue in 3% acetic acid solution pH 2.5.

### 2.6. Quantitative Reverse Transcriptase Polymerase Chain Reaction (qRT-PCR)

After a 21-day differentiation period, total RNA was isolated from undifferentiated MSPCs (ctrl) and osteogenically differentiated MSPCs using the RNeasy Mini Kit and a DNase-I treatment, according to the manufacturer’s manual (Qiagen, Hilden, Germany). Using the iScript cDNA Synthesis Kit, (BioRad Laboratories Inc., Hercules, CA, USA), 1 µg of RNA was reverse transcribed using a mixture of oligo(dT) and random hexamer primers. The amplification was performed with the SsoAdvanced Universal SYBR Green Supermix, whereby each qRT-PCR run from a standard 3-step PCR temperature protocol (annealing temperature of 60 °C); measured with the CFX96 Touch (BioRad Laboratories Inc., Hercules, CA, USA). A melting curve protocol was used to confirm the individual gene-specific peaks and to detect primer dimerization. Relative quantification of expression levels was obtained by the ΔΔCt method based on the geometric mean of the internal controls TBP (TATA-box binding protein) and RPLP0 (ribosomal protein, lateral stalk, subunit P0), respectively. The expression levels (Ct) of the target genes were normalized to the reference genes (ΔCt), and the difference between the ΔCt value of the test sample and the ΔCt of the control sample gave the ΔΔCt value. Finally, the expression ratio was expressed as 2^ΔΔCt^. The following QuantiTect primer assays (Qiagen, Hilden, Germany) were used for qRT-PCR: alkaline phosphatase (ALP), osteopontin (SPP1), osteonectin (SPARC), osteocalcin (OC), and the integrin subunits ITGα5 and ITGβ1.

### 2.7. xMAP Human Bone Metabolism Magnetic Bead Panel

Using the Luminex^®^ xMAP^®^ platform in a magnetic bead format, we simultaneously analyzed the following targets from cell culture supernatant samples: adrenocorticotropic hormone (ACTH), Dickkopf-related protein 1 (DKK1), interleukin-6 (IL6), insulin, leptin, tumor necrosis factor α (TNFα), osteocalcin (OC), osteopontin (SPP1), osteoprotegerin (OPG), sclerostin (SOST), interleukin-1β (IL-1β), parathyroid hormone (PTH), and fibroblast growth factor 23 (FGF23), according to the manufacturer’s instructions. There was no cross-reactivity between the antibodies for an analyte and any of the other analytes in this panel.

### 2.8. Statistical Analysis

The unpaired student’s t-test and the exact Wilcoxon test with the PASW statistics software 18 (IBM Corporation, Somers, NY, USA) was used to evaluate the differences between the groups. Two-sided P-values were defined as statistically significant (*p* < 0.001***; *p* < 0.01**; *p* < 0.05*). The graphical representations were created with SigmaPlot^®^ 14.0 (Systat Software Inc., San Jose, CA, USA).

## 3. Results

### 3.1. Surface Characteristics

**Figure 1:**
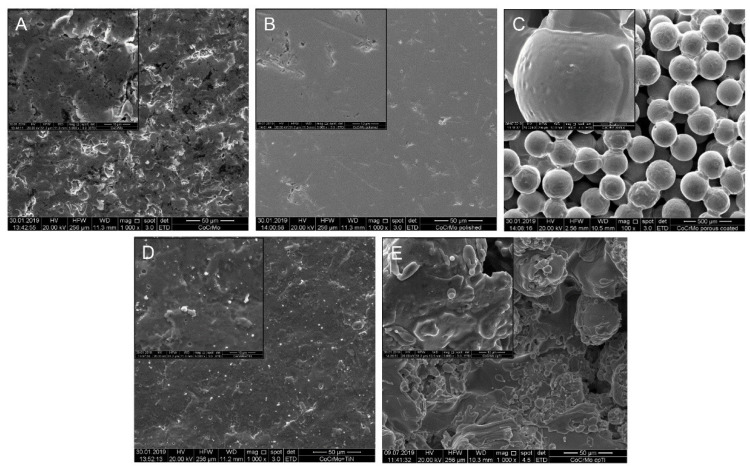
Surface characteristics, adapted from [21]. Scanning electron microscopy (SEM) of the cobalt chromium molybdenum (CoCrMo) alloy surface modifications of (**A**) uncoated CoCrMo, (**B**) polished surface (both 1000× and as an insert 5000× magnification), (**C**) porous coated surface (100× and 1000× magnification), (**D**) titanium nitride (TiN), and (**E**) pure titanium (cpTi) coated surfaces (1000× and 5000× magnification).

**Figure 2:**
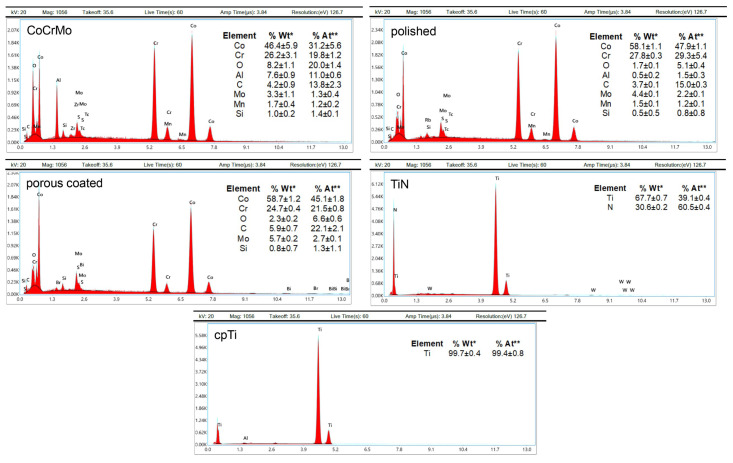
Energy-dispersive X-ray (EDX) analysis and elemental quantification, adapted from [21]. EDX analysis exhibited no differences between the uncoated CoCrMo, the polished, and the porous coated surface with regard to the composition of the chemical elements, whereas the TiN and cpTi coatings fundamentally changed the surface quality. %Wt*: Percentage of the total weight of the sample; %At**: Percentage in relation to the atomic weight of the sample (mean ± SD; n = 3).

The surface characterization results presented in this section are derived from a previous study [21]. The modified morphology of the discs was determined using scanning electron microscopy (SEM). The microscopic SEM images showed significant differences in the morphology of the uncoated CoCrMo slices (magnification 1000× and as insert 5000×) compared to the respective modifications (Figure 1). The polished surface showed only very few structures, whereas the spherical structures of the porous coated surface were particularly well visible at 100× and 1000× magnification. The size of the spheroids was between 250–355 µm. Macroscopically, the TiN coating showed a metallic, golden yellow appearance, with the coating adhering particularly well to the implant. The surface of the TiN coating was slightly roughened (Ra < 0.05 µm). The special coating process of vacuum plasma spraying with pure titanium (cpTi) resulted in a structured surface with rounded elements (both 1000× and 5000× magnification). Elemental analysis of each substrate was performed using EDX (Figure 2). The EDX analysis and its quantification showed no differences between the uncoated CoCrMo, the polished, and the porous coated surface regarding the composition of the chemical elements. However, a fundamental change in surface quality could be detected for the TiN and cpTi coating.

### 3.2. MSPC Characterization and Multilineage Differentiation Analysis

**Figure 3:**
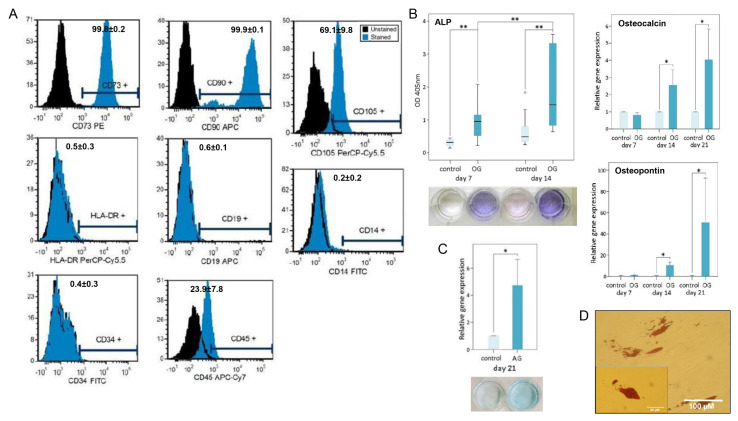
Mesenchymal stem and progenitor cells (MSPC) characterization and multilineage differentiation analysis. The specific characteristics of the human primary MSPCs used were demonstrated according to (**A**) the positive expression of CD73, CD90, CD105, and the negative expression of CD14, CD19, CD34, CD45, and HLA-DR. The percentage of positive stained cells are presented (mean ± SD; n = 3). The multilineage differentiation potential was proved for all primary cultures by (**B**) the relative expression of alkaline phosphatase (ALP), osteocalcin, and osteopontin for the osteogenic differentiation (mean ± SD; n = 7; measured in quadruplicate), (**C**) Alcian blue staining and the expression of aggrecan for the chondrogenic differentiation (mean ± SD; n = 7; measured in quadruplicate), and (**D**) the oil red O staining of lipid droplets for the adipogenic lineage. One representative picture is shown. **: *p* < 0.01; *: *p* < 0.05.

Within 4–8 days, cells corresponding to the morphological characteristics of human primary MSPCs (mononuclear, fibroblast-like, spindle-shaped, plastic adhesive) could be isolated from all bone samples. In the FACS analyses, the MSPCs showed a positive expression of CD73 (99.8 ± 0.2%), CD90 (99.9 ± 0.1%), and CD105 (69.1 ± 9.8%) of gated cells. The negative expression of CD14 (0.2 ± 0.2%), CD19 (0.6 ± 0.1%), CD34 (0.4 ± 0.3%), CD45 (23.9 ± 7.8%), and HLA-DR (0.5 ± 0.3%) confirmed the phenotype of MSPCs according to the criteria of the International Society for Cellular Therapy [22] (Figure 3A). As a further indicator, MSPCs were successfully differentiated in the direction of the osteogenic, chondrogenic, and adipogenic line. With a significant increase (*p* < 0.001) over time, ALP expression during osteogenic differentiation was demonstrated on days 7 and 14. No expression of this enzyme was observed in the undifferentiated negative controls (Figure 3B). The osteogenic late stage markers osteocalcin and osteopontin were analyzed by relative gene expression after 7, 14, and 21 days of osteogenic differentiation (OG) (Figure 3B). The increase in blue coloration, which is due to the interaction of the cationic dye Alcian blue with acidic glycosaminoglycans, was observed in chondrogenically differentiated MSPCs compared to undifferentiated controls. Analysis of the expression of aggrecan demonstrated a 4.7-fold increase (*p* < 0.05) as a result of chondrogenic differentiation (Figure 3C). The adipogenic cell differentiation was demonstrated by the formation of lipid vacuoles, which were made visible by oil red O staining on day 21 (Figure 3D). This demonstrated the multilineage ability of the cells, which could be explicitly characterized as MSCPs.

### 3.3. Expression of Osteogenic Markers on Different CoCrMo Surface Modifications

**Figure 4:**
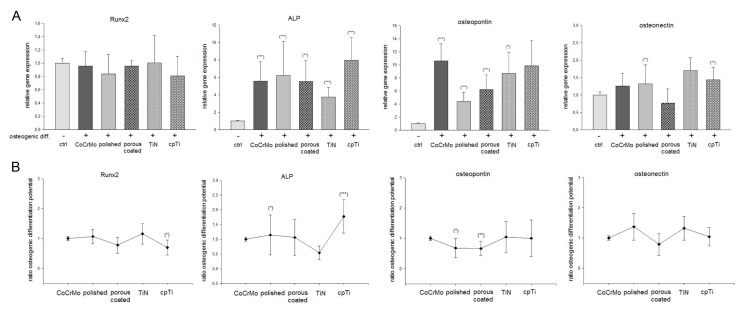
Expression of osteogenic markers on different CoCrMo surface modifications. MSPCs were seeded on the different material discs and the cells were osteogenically differentiated over three weeks. After RNA isolation, qRT-PCR of the relative expression of Runt-related transcription factor 2 (Runx2), alkaline phosphatase (ALP), osteopontin, and osteonectin were performed. Undifferentiated MSPCs (ctrl) served as references (ratio = 1) (mean ± SD; n = 7, measured in triplicate). (**A**) Represents the changes of important osteogenic markers by differentiation on the CoCrMo material modifications and (**B**) displays the changes within the material groups. ***: *p* < 0.001; **: *p* < 0.01; *: *p* < 0.05.

We investigated the influence of CoCrMo surface modifications on the potential of osteogenic differentiation. MSPCs were seeded on the different material discs and the cells were osteogenically differentiated over 21 days. After RNA isolation, RT-qPCR of the relative expression of Runx2, ALP, osteopontin, and osteonectin were performed. Undifferentiated MSPCs (ctrl) served as references (ratio = 1). After osteogenic differentiation, Runx2 expression did not show any significant change, whereas ALP expression increased significantly in all groups about 5-fold compared to the undifferentiated controls (Figure 4A). While MSPCs on TiN-coated discs showed an increase of 3.2 ± 0.5, the strongest increase in ALP expression to 8.2 ± 2.2 was observed on cpTi-coated discs. This development is also reflected in the comparison of the material surfaces.

In relation to the osteogenic differentiation potential of the uncoated CoCrMo surface, the polished surface and cpTi coated surface significantly increased ALP expression (Figure 4B). With respect to osteopontin expression, a 10-fold higher expression was observed in the osteogen differentiated groups of CoCrMo, TiN, and cpTi (Figure 4A). In relation to the CoCrMo comparison group, the polished and porous coated groups performed slightly worse (Figure 4B). Almost no differences could be observed in osteonectin expression. Only the porous coated group showed a reduced expression of this osteogenic marker. All values (mean ± SD; n = 7, measured in triplicate) and significances are listed in Table 1.

### 3.4. The Role of Integrin α5β1 Subunits on Different CoCrMo Surface Modifications

**Figure 5:**
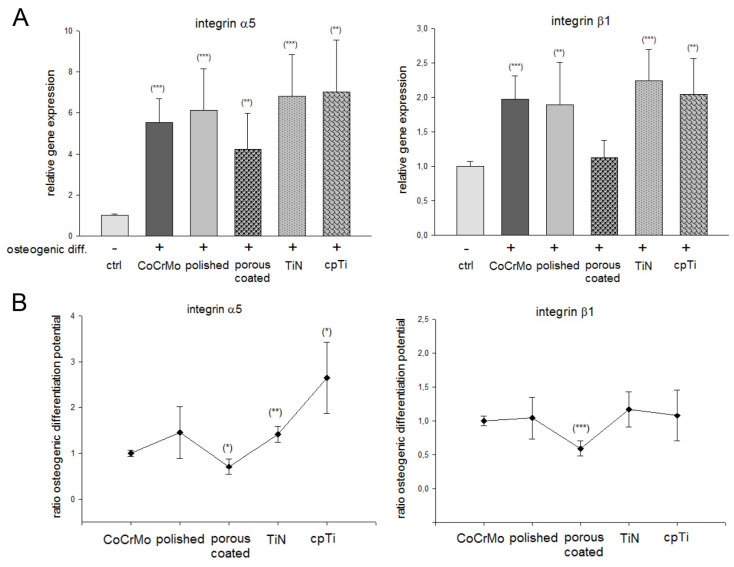
The role of integrin α5β1 subunits on different CoCrMo surface modifications. The relative mRNA expression of integrin α5 and β1 subunits are shown compared to the undifferentiated controls. (**A**) Represents the changes by differentiation on the CoCrMo material modifications and (**B**) displays the changes within the material groups (mean ± SD; n = 7, measured in triplicate). ***: *p* < 0.001; **: *p* < 0.01; *: *p* < 0.05.

Under the same conditions as for the analysis of the osteogenic markers, the MSPCs were seeded on the material discs. The relative mRNA expression showed a 4–7-fold increased expression of integrin α5 and a 1.2–2.4-fold increased expression of integrin β1 compared to the undifferentiated controls (Figure 5A). In relation to the CoCrMo control group, the porous coated surface showed the lowest expression consistent. The coating with pure titanium (cpTi) specifically increased the expression of integrin α5 (Figure 5B). All values (mean ± SD; n = 7, measured in triplicates) are also listed in Table 1.

### 3.5. Expression of Bone Biology Markers on Different CoCrMo Surface Modifications

**Figure 6:**
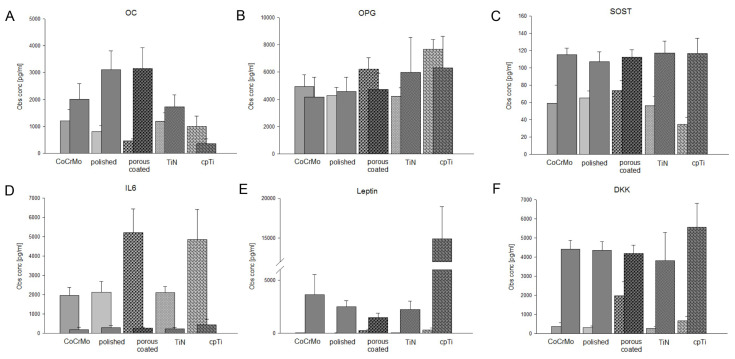
Expression of bone biology markers on different CoCrMo surface modifications. Using the Luminex^®^ xMAP^®^ platform, we simultaneously analyzed the expression of 13 important bone related markers. The expression of (**A**) osteocalcin (OC), (**B**) osteoprotegerin (OPG), (**C**) sclerostin (SOST), (**D**) interleukin-6 (IL6), (**E**) leptin, and (**F**) Dickkopf-related protein 1 (DKK1) are presented with bright bars (undifferentiated controls) and dark, laterally offset bars (osteogenic differentiated groups) (mean ± SD; n = 5, measured in duplicate). ACTH, insulin, TNFα, IL1β, PTH, and FGF23 were out of range.

Next, we measured the expression of important bone related proteins and cytokines. The expression of ACTH, insulin, TNFα, IL1β, PTH, and FGF23 were below the detection limit. The bright, left-sided bars represent the undifferentiated controls, and the dark, laterally offset bars represent the respective osteogenic differentiated groups (Figure 6). To make it easier to understand, the different significances are not shown in the figure. All values (mean ± SD; n = 5, measured in duplicate) and significances are listed in Table 2.

With the exception of the cpTi coated surfaces, all osteogenic groups showed significant increases in OC expression (Figure 6A), whereas the OPG expression showed no significant differences (Figure 6B). Sclerostin (SOST) expression also increased significantly due to osteogenic differentiation (Figure 6C). Interestingly, differences between the different surfaces are more likely to be observed in the undifferentiated MSPCs. A high increase in IL6 expression in undifferentiated MSPCs on the porous coated and cpTi discs was measured (Figure 6D). The leptin release, on the other hand, increased highly significantly due to osteogenic differentiation (Figure 6E), which was especially high in the cpTi samples. Dickkopf-related protein 1 (DKK) increased due to osteogenic differentiation, but showed no differences between the different surfaces (Figure 6F).

## 4. Discussion

The mechanical stability of CoCrMo and its resistance to wear make it a widely used material for orthopedic applications. However, the biocompatibility of other metals such as titanium is lacking [23]. In recent years, intensive research has been carried out on modifications of the material surface in order to increase the attractiveness of the material for cells and thus optimize the integration of the material into the surrounding tissue. One such modification is a change in the surface roughness and topography of the prosthesis material. Dalby et al. revealed that MSPCs can be affected by topographical features present on a material surface [24]. With the support of a commercial manufacturer of orthopedic implants, surface modifications were produced using polishing, sintering, and coating processes. We investigated the osteogenic differentiation potential and the expression of bone related markers of primary human MSPCs on different CoCrMo alloy surface modifications: a titanium nitride (TiN) coating, a porous coated surface, a polished surface, and a coating with pure titanium (cpTi). The different surfaces were represented by SEM and elemental analysis of each substrate was performed using EDX.

Human primary MSPCs were isolated from tissue samples of spongiosa bone and characterized by the positive and negative expression of specific surface markers and the multilineage differentiation. Based on the Runt-related transcription factor 2 (Runx2), we analyzed the cascade of osteogenic differentiation markers using RT-qPCR. The Runx2 transcript has the ability to facilitate the convergence of numerous osteogenic signaling pathways and stimulate genes like osteopontin, osteonectin, osteocalcin, and ALP [25]. Runx2 expression did not change during in vitro differentiation, while the concentrations of downstream genes such as ALP were dramatically increased. Corresponding studies have confirmed these observations [26] and our data showed the same effect. With respect to the different surfaces, the cpTi coating showed a significantly lower expression compared to the uncoated CoCrMo comparison group. ALP serves as an early indicator of differentiation, whereby the ALP-mRNA level rises with the time course of osteogenic differentiation [27,28]. The osteogenic differentiation caused the values of our MSPCs to increase 4–7-fold. The polished and cpTi coated surfaces showed significant increases compared to the control group.

Osteopontin, a secreted adhesive glycophosphoprotein, plays a key role within bone tissue for cell adhesion, migration, and survival [29]. The expression increased significantly due to the osteogenic differentiation. Compared to the CoCrMo control group, however, only minor differences were observed in the polished and porous coated surfaces. Similar data were found with osteopontin. Osteocalcin has a key role in the differentiation of osteoblast precursor cells [30,31]. Integrin interactions represent a key role in the regulation of the osteocalcin gene. The blocking of integrin-ECM interactions subsequently blocks the ascorbic acid-dependent OSE2 activation. Klontzas et al. [20] demonstrated that glycine–histidine–lysine peptides, which are fragments of osteonectin, significantly increased the expression of integrin β1 in cord blood MSPCs.

We investigated the expression of integrin α5β1 to get a possible conclusion on the influence of the modified surfaces. Cell–material contact is of great importance for the regulation of MSPCs. The expression of the integrin α5β1 is a central mechanism by which a material surface can improve the osteogenesis [19,32,33]. Our data revealed that osteogenic differentiation increased the expression of the α5 subunit by a factor of 4–6 and the β1 subunit by a factor of 1.5–2. TiN and cpTi surfaces stood out positively. The lowest expression of α5β1was shown on the porous coating surface. These results are consistent with those of Logan et al. [34], where smooth surfaces with a R_a_ value of about 1 increased the biocompatibility and adhesion of CoCrMo materials. While the TiN coating was within this range, our cpTi surface had a significantly higher value and strengthened the adhesion properties compared to the uncoated CoCrMo surface.

Bone biology is a dynamic process of ongoing bone deposition and resorption. Osteoblasts differ from mesenchymal progenitor cells in their biological development and pass through different stages of development, which are regulated differently [35,36]. In order to analyze this regulation and the influence of surface modifications, we performed a Luminex^®^ based 13plex assay. ACTH, insulin, TNFα, IL-1β, PTH, and FGF23 were below the detection limit. OC expression showed differences between the different surfaces, both in the undifferentiated and in the osteogenically differentiated MSPCs, whereas these could not be observed in OPG, a member of the TNF super-family, and SOST. IL6 expression was significantly upregulated in undifferentiated MSPCs on porous coated and cpTi surfaces. During the aseptic loosening of endoprosthetic implants, metal particles as well as their corrosion products have been shown to cause a biological reaction. Cobalt–chromium ions cause reduced OPG protein synthesis and increased secretion of inflammatory parameters such as IL6 [36,37,38]. Leptin was initially best known for its role in energy homeostasis, but also plays a major role in bone biology [39]. Leptin and the corresponding receptor are expressed in MSPCs and might act as an autocrine factor in the osteogenesis of MSPCs and bone remodeling. In particular, the cpTi coating seemed to have a big influence on leptin expression. However, Zhou et al. demonstrated in a fracture model that 85% of osteoblasts were derived from leptin receptor-positive MSPCs after eight weeks [40]. This proves the importance of these autocrine factors in bone biology. In order to fully understand these complex processes, further experiments must be carried out.

## 5. Conclusions

With regard to the osteogenic differentiation potential, the coating with pure titanium (cpTi) in particular had a positive effect, whereas the porous coated surface showed poor results. The expression of integrin α5β1, which is particularly important for cell–material contact, also reflected this process. In the dynamic process of bone biology, MSPCs cultured and differentiated on cpTi showed significant upregulation of IL6 and leptin.

## Figures and Tables

**Table 1:** Osteogenic differentiation potential. All relative expression values of the osteogenic differentiation experiments are listed (mean ± SD; n = 7, measured in triplicates).

**Gene.**	**Ctrl**	**Osteogenic Differentiated**				
**ad Figure 3**		**CoCrMo**	**Polished**	**Porous**	**TiN**	**cpTi**
**Runx2** (Figure 3A)	1 ± 0.1	0.95 ± 0.2	0.84 ± 0.3	0.96 ± 0.1	1.00 ± 0.4	0.81 ± 0.3
**Runx2** (Figure 3B)		1 ± 0.1	1.07 ± 0.2	0.77 ± 0.3	1.16 ± 0.3	0.69 ± 0.2 *
**ALP** (Figure 3A)	1 ± 0.1	5.17 ± 2.4 ***	5.92 ± 4.1 **	5.66 ± 3.3 ***	3.93 ± 1.8 ***	7.74 ± 2.8 ***
**ALP** (Figure 3B)		1 ± 0.1	1.62 ± 0.4*	1.26 ± 0.7	1.24 ± 0.3	1.93 ± 0.5 ***
**Osteopontin** (Figure 3A)	1 ± 0.1	10.63 ± 4.0 ***	4.38 ± 1.4 ***	5.73 ± 4.2 *	8.74 ± 3.2 ***	9.38 ± 5.6
**Osteopontin** (Figure 3B)		1 ± 0.1	0.68 ± 0.3 *	0.66 ± 0.2 **	1.05 ± 0.5	0.84 ± 0.7
**Osteonectin** (Figure 3A)	1 ± 0.1	1.26 ± 0.3	1.33 ± 0.5	0.77 ± 0.4	1.74 ± 0.4 **	1.44 ± 0.3 **
**Osteonectin** (Figure 3B)		1 ± 0.1	1.37 ± 0.5	0.79 ± 0.4	1.32 ± 0.4	1.04 ± 0.3
	**Ctrl**	**Osteogenic Differentiated**				
**ad Figure 4**		**CoCrMo**	**Polished**	**Porous**	**TiN**	**cpTi**
**Integrin α5** (Figure 4A)	1 ± 0.1	5.53 ± 1.2 ***	6.13 ± 2.0 ***	4.22 ± 1.8 **	6.83 ± 2.0 ***	7.03 ± 2.5 **
**Integrin α5** (Figure 4B)		1 ± 0.1	1.41 ± 0.6	0.69 ± 0.2 *	1.35 ± 0.3 *	2,65 ± 0.8
**Integrin β1** (Figure 4A)	1 ± 0.1	1.97 ± 0.3 ***	1.89 ± 0.6 **	1.17 ± 0.2	2.25 ± 0.4 ***	2.16 ± 0.5 **
**Integrin β1** (Figure 3B)		1 ± 0.1	1.04 ± 0.3	0.59 ± 0.11 ***	1.17 ± 0.3	1.07 ± 0.3

***: *p* < 0.001; **: *p* < 0.01; *: *p* < 0.05.

**Table 2:** Bone biology data. Observed concentration (Obs conc.) values of undifferentiated (undiff) and osteogenic differentiated (OG) MSPCs were listed. Significant differences between the undifferentiated and differentiated groups are displayed with an asterix (*), the differences between the original CoCrMo surface and the surface modifications (SM) are represented by rhombus (#) (mean ± SD; n = 5, measured in duplicates).

	pg/mL				
Gene	CoCrMo	Polished	Porous	TiN	cpTi
**OC** undiff	1200 ± 419	805 ± 218	467 ± 69	1195 ± 306	999 ± 379
**OC** OG	2003 ± 591	3158 ± 775 *	1727 ± 447 *	3117 ± 699 *	354 ± 184 *
SM undiff.		n.s.	#	n.s.	n.s.
SM OG		n.s.	n.s.	n.s.	##
**OPG** undiff	4934 ± 879	4279 ± 593	6221 ± 840	4224 ± 639	7674 ± 710
**OPG** OG	4176 ± 1462	4740 ± 1178	5985 ± 2565	4583 ± 1043	6315 ± 2307
SM undiff.		n.s.	n.s	n.s.	##
SM OG		n.s.	n.s	n.s.	n.s.
**SOST** undiff	58.8 ± 21	65.1 ± 8	73.6 ± 11.8	56.2 ± 10.5	35.0 ± 7.9
**SOST** OG	115 ± 7.9 *	112.5 ± 8.6 ***	117.1 ± 13.8 *	107.3 ± 11.3 **	116.7 ± 17.6 **
SM undiff.		n.s.	#	n.s.	n.s.
SM OG		n.s.	n.s.	n.s.	n.s.
**IL6** undiff	1957 ± 426	2114 ± 575	5208 ± 1230	2099 ± 324	4857 ± 1556
**IL6** OG	187 ± 128 ***	262 ± 57 *	218 ± 76 *	295 ± 91 **	435 ± 296 *
SM undiff.		n.s.	#	n.s.	#
SM OG		n.s.	n.s.	n.s.	n.s.
**Leptin** undiff	27.3 ± 4.5	17.7 ± 4.6	267 ± 107	33 ± 12.3	313 ± 175
**Leptin** OG	3642 ± 1877 ***	1478 ± 405 **	2230 ± 811 **	2498 ± 549 ***	14911 ± 4053 ***
SM undiff.		#	#	n.s.	##
SM OG		n.s.	n.s.	n.s.	##
**DKK** undiff	371 ± 196	311 ± 79	1978 ± 737	266 ± 106	659 ± 232
**DKK** OG	4410 ± 468 ***	4179 ± 449 ***	3816 ± 1482	4350 ± 641 ***	5557 ± 1266 **
SM undiff.		n.s.	#	n.s.	n.s.
SM OG		n.s.	n.s.	n.s.	n.s.

***: *p* < 0.001; **,##: *p* < 0.01; *,#: *p* < 0.05; n.s.: not significant.
