# Correction: Lohberger et al. Cobalt Chromium Molybdenum Surface Modifications Alter the Osteogenic Differentiation Potential of Human Mesenchymal Stem Cells. *Materials* 2020, *13*, 4292

**DOI:** 10.3390/ma18122873

**Published:** 2025-06-18

**Authors:** Birgit Lohberger, Nicole Eck, Dietmar Glaenzer, Helga Lichtenegger, Leon Ploszczanski, Andreas Leithner

**Affiliations:** 1 Department of Orthopedics and Trauma, Medical University Graz, 8036 Graz, Austria; nicole.eck@medunigraz.at (N.E.); dietmar.glaenzer@medunigraz.at (D.G.); andreas.leithner@medunigraz.at (A.L.); 2 Department of Material Sciences and Process Engineering, Institute of Physics and Materials Science, University of Natural Resources and Life Sciences, 1160 Vienna, Austria; helga.lichtenegger@boku.ac.at (H.L.); leon.ploszczanski@boku.ac.at (L.P.)

In the original publication [1], the paper “Lohberger, B.; Stuendl, N.; Glaenzer, D.; Rinner, B.; Donohue, N.; Lichtenegger, H.C.; Ploszczanski, L.; Leithner, A. CoCrMo surface modifications affect biocompatibility, adhesion, and inflammation in human osteoblasts. *Sci. Rep.* **2020**, *10*, 1682” was not cited. The citation has now been inserted in the captions of Figure 1 and Figure 2 as reference [21]. Additionally, a new sentence has been inserted in Section 2.2 Scanning Electron Microscopy (SEM) and Section 3.1 Surface Characteristics. The updated text should read as follows:

Caption of Figure 1:

**Figure 1.** Surface characteristics, adapted from [21]. Scanning electron microscopy (SEM) of the cobalt chromium molybdenum (CoCrMo) alloy surface modifications of (**A**) uncoated CoCrMo, (**B**) polished surface (both 1000× and as an insert 5000× magnification), (**C**) porous coated surface (100× and 1000× magnification), (**D**) titanium nitride (TiN), and (**E**) pure titanium (cpTi) coated surfaces (1000× and 5000× magnification).

Caption of Figure 2:

**Figure 2.** Energy-dispersive X-ray (EDX) analysis and elemental quantification, adapted from [21]. EDX analysis exhibited no differences between the uncoated CoCrMo, the polished, and the porous coated surface with regard to the composition of the chemical elements, whereas the TiN and cpTi coatings fundamentally changed the surface quality. %Wt*: Percentage of the total weight of the sample; %At**: Percentage in relation to the atomic weight of the sample (mean ± SD; n = 3).

Section 2.2. Scanning Electron Microscopy (SEM):

**SEM and EDX measurements have been performed in a previous study [21], as briefly described follows.** The SEM images were taken with the FEI Quanta 250 FEG (Thermo Fisher Scientific, Hillsboro, OR, USA) under high vacuum conditions and 20 kV high voltage. The micrographs were taken with an Everhart–Thornley detector in secondary electron (SE) mode. To ensure sufficient electrical conductivity, the surfaces were sputter-coated with a 10 nm thin gold layer. The energy-dispersive X-ray spectroscopy (EDX) measurements were performed for 60 s, 20 kV high tension, and a spot size of 4.5 with a 30 mm^2^ Octane Elect Plus Silicon Drift Detector by EDAX Ametek (Berwyn, PA, USA) and APEX standard software V1.3.1 from 6 July, 2019.

Section 3.1. Surface Characteristics:

**The surface characterization results presented in this section are derived from a previous study [21].** The modified morphology of the discs was determined using scanning electron microscopy (SEM). The microscopic SEM images showed significant differences in the morphology of the uncoated CoCrMo slices (magnification 1000× and as insert 5000×) compared to the respective modifications (Figure 1). The polished surface showed only very few structures, whereas the spherical structures of the porous coated surface were particularly well visible at 100× and 1000× magnification. The size of the spheroids was between 250–355 μm. Macroscopically, the TiN coating showed a metallic, golden yellow appearance, with the coating adhering particularly well to the implant. The surface of the TiN coating was slightly roughened (Ra < 0.05 µm). The special coating process of vacuum plasma spraying with pure titanium (cpTi) resulted in a structured surface with rounded elements (both 1000× and 5000× magnification). Elemental analysis of each substrate was performed using EDX (Figure 2). The EDX analysis and its quantification showed no differences between the uncoated CoCrMo, the polished, and the porous coated surface regarding the composition of the chemical elements. However, a fundamental change in surface quality could be detected for the TiN and cpTi coating.

With this correction, the order of some references has been adjusted accordingly. The authors state that the scientific conclusions are unaffected. This correction was approved by the Academic Editor. The original publication has also been updated.
